# Supplementary material for: The Foliar Application of Rice Phyllosphere Bacteria induces Drought-Stress Tolerance in Oryza sativa (L.)
Source: Plants (Basel). 2021 Feb 18;10(2):387. doi: 10.3390/plants10020387 (PMC7923115; doi:10.3390/plants10020387)
Supplement: Supplementary file 1 [file plants-10-00387-s001.pdf]

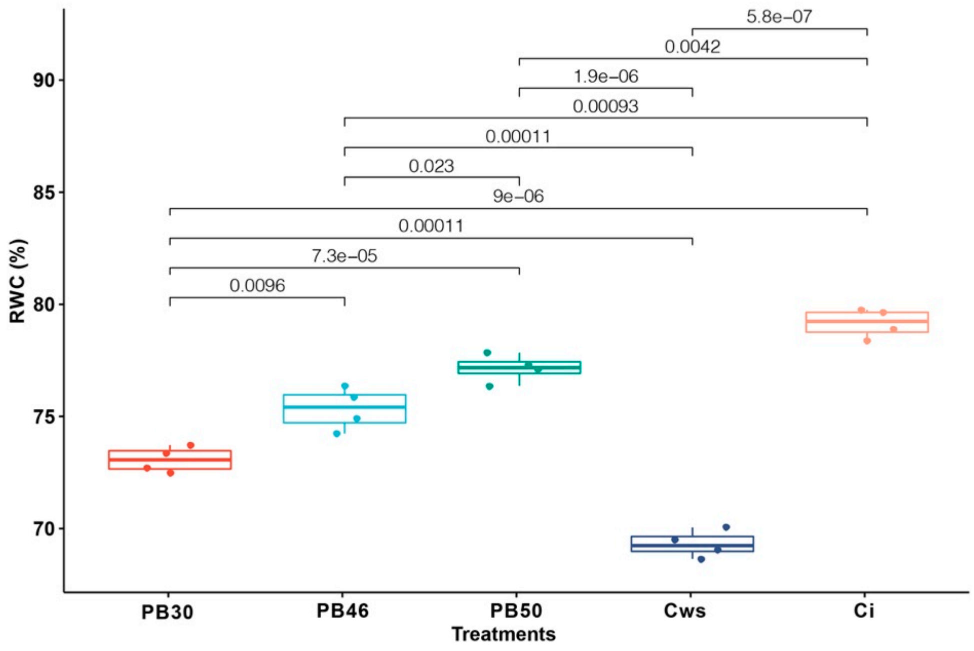

Figure S1: Relative water content (RWC) in rice leaves of different treatment, as well as statistically (ANOVA) significant differences between treatments (ANOVA, p<0.000) after 10 days of drought stress.

Data shown are mean and standard deviation (n = 4). Numbers given above the brackets are P-values indicating statistically significant differences between two treatments according to the Duncan`s test. PB3, drought stress with *B. endophyticus* PB3 foliar spray; PB46, drought stress with *B. altitudinis* PB46 foliar spray; PB50, drought stress with *B. megaterium* PB50 foliar spray; Cws, drought stress control and Ci, irrigated control.

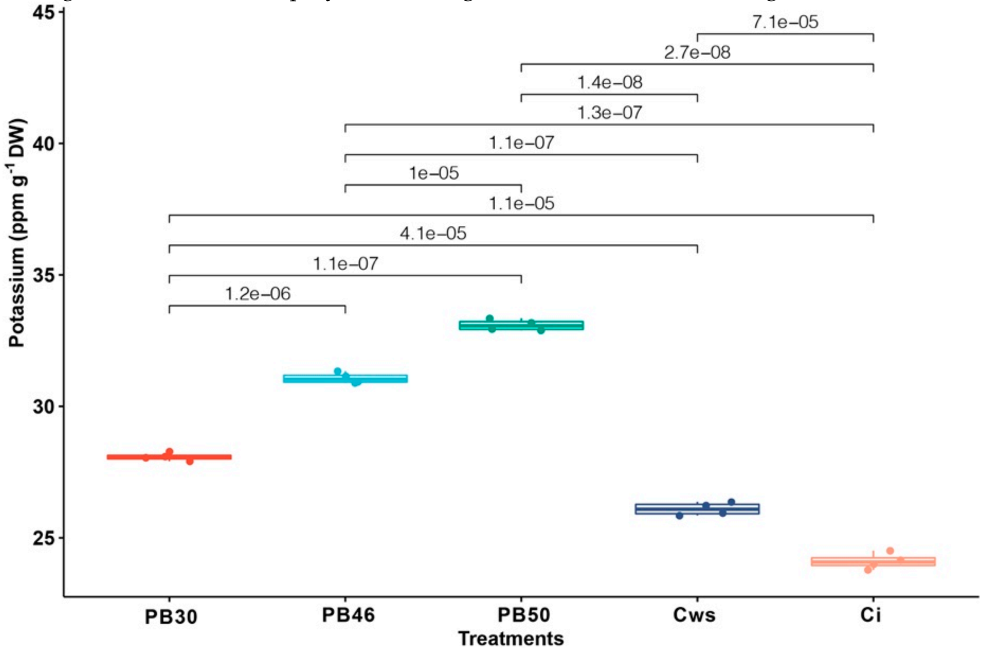

Figure S2: Potassium content in rice leaves of different treatment, as well as statistically (ANOVA) significant differences between treatments (ANOVA, p<0.000) after 10 days of drought stress.

Data shown are mean and standard deviation (n = 4). Numbers given above the brackets are P-values indicating statistically significant differences between two treatments according to the Duncan`s test. PB3, drought stress with *B. endophyticus* PB3 foliar spray; PB46, drought stress with *B. altitudinis* PB46 foliar spray; PB50, drought stress with *B. megaterium* PB50 foliar spray; Cws, drought stress control and Ci, irrigated control.

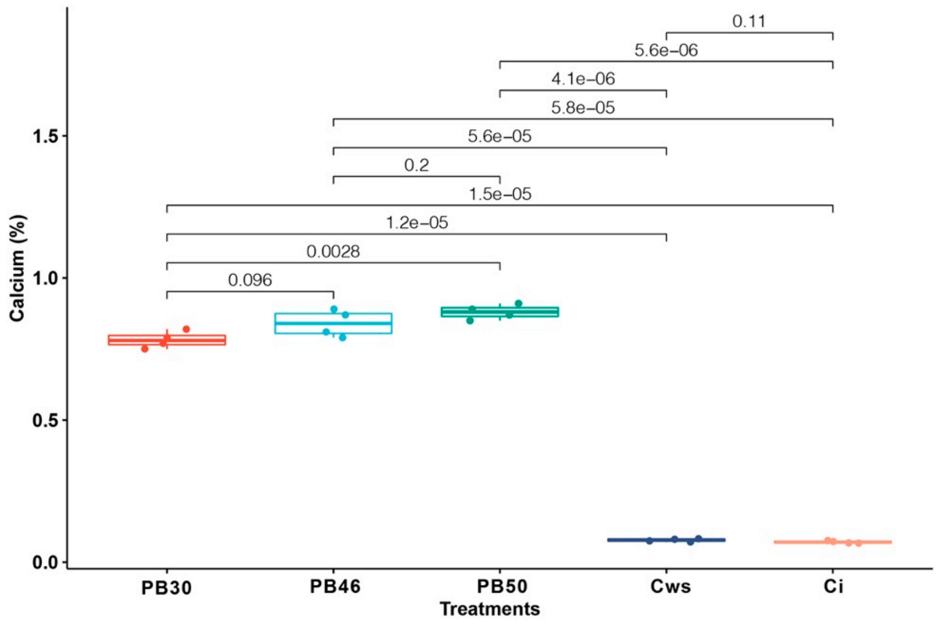

Figure S3: Calcium content in rice leaves of different treatment, as well as statistically (ANOVA) significant differences between treatments (ANOVA, p<0.000) after 10 days of drought stress.

Data shown are mean and standard deviation (n = 4). Numbers given above the brackets are P-values indicating statistically significant differences between two treatments according to the Duncan`s test. PB3, drought stress with *B. endophyticus* PB3 foliar spray; PB46, drought stress with *B. altitudinis* PB46 foliar spray; PB50, drought stress with *B. megaterium* PB50 foliar spray; Cws, drought stress control and Ci, irrigated control.

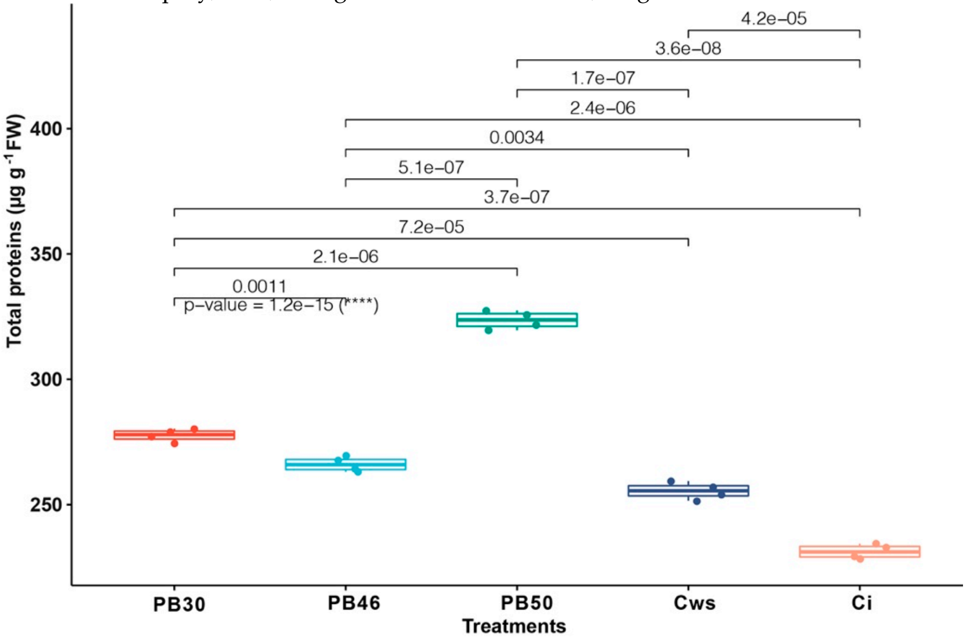

Figure S4: Total proteins in rice leaves of different treatment, as well as statistically (ANOVA) significant differences between treatments (ANOVA, p<0.000) after 10 days of drought stress.

Data shown are mean and standard deviation (n = 4). Numbers given above the brackets are P-values indicating statistically significant differences between two treatments according to the Duncan`s test. PB3, drought stress with *B. endophyticus* PB3 foliar spray; PB46, drought stress with *B. altitudinis* PB46 foliar spray; PB50, drought stress with *B. megaterium* PB50 foliar spray; Cws, drought stress control and Ci, irrigated control.

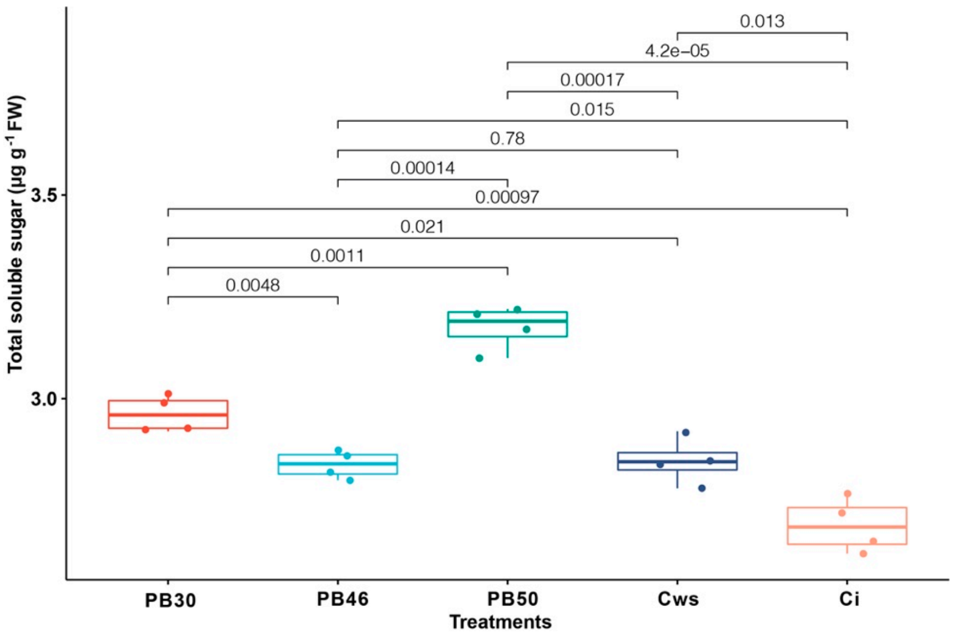

Figure S5: Total soluble sugars in rice leaves of different treatment, as well as statistically (ANOVA) significant differences between treatments (ANOVA, p<0.000) after 10 days of drought stress.

Data shown are mean and standard deviation (n = 4). Numbers given above the brackets are P-values indicating statistically significant differences between two treatments according to the Duncan`s test. PB3, drought stress with *B. endophyticus* PB3 foliar spray; PB46, drought stress with *B. altitudinis* PB46 foliar spray; PB50, drought stress with *B. megaterium* PB50 foliar spray; Cws, drought stress control and Ci, irrigated control.

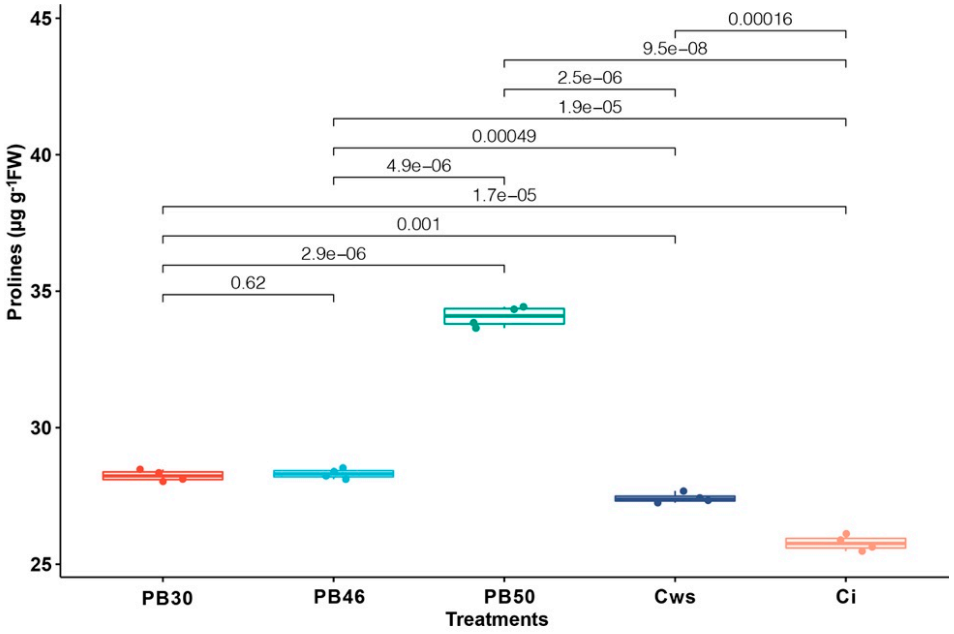

Figure S6: Total prolines in rice leaves of different treatment, as well as statistically (ANOVA) significant differences between treatments (ANOVA, p<0.000) after 10 days of drought stress.

Data shown are mean and standard deviation (n = 4). Numbers given above the brackets are P-values indicating statistically significant differences between two treatments according to the Duncan`s test. PB3, drought stress with *B. endophyticus* PB3 foliar spray; PB46, drought stress with *B. altitudinis* PB46 foliar spray; PB50, drought stress with *B. megaterium* PB50 foliar spray; Cws, drought stress control and Ci, irrigated control.

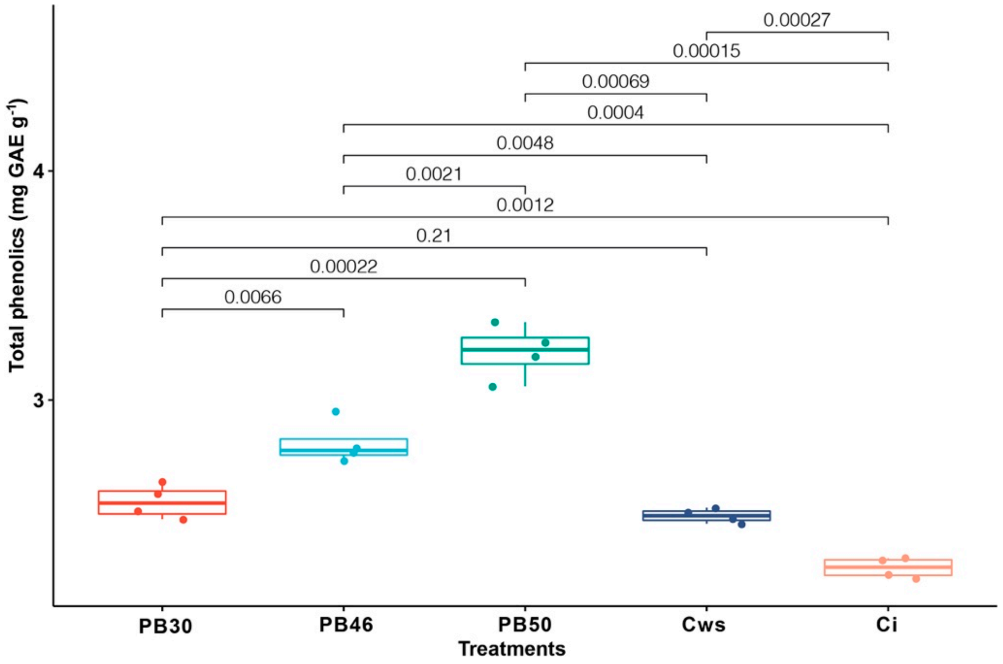

Figure S7: Total phenolics in rice leaves of different treatment, as well as statistically (ANOVA) significant differences between treatments (ANOVA, p<0.000) after 10 days of drought stress.

Data shown are mean and standard deviation (n = 4). Numbers given above the brackets are P-values indicating statistically significant differences between two treatments according to the Duncan`s test. PB3, drought stress with *B. endophyticus* PB3 foliar spray; PB46, drought stress with *B. altitudinis* PB46 foliar spray; PB50, drought stress with *B. megaterium* PB50 foliar spray; Cws, drought stress control and Ci, irrigated control.

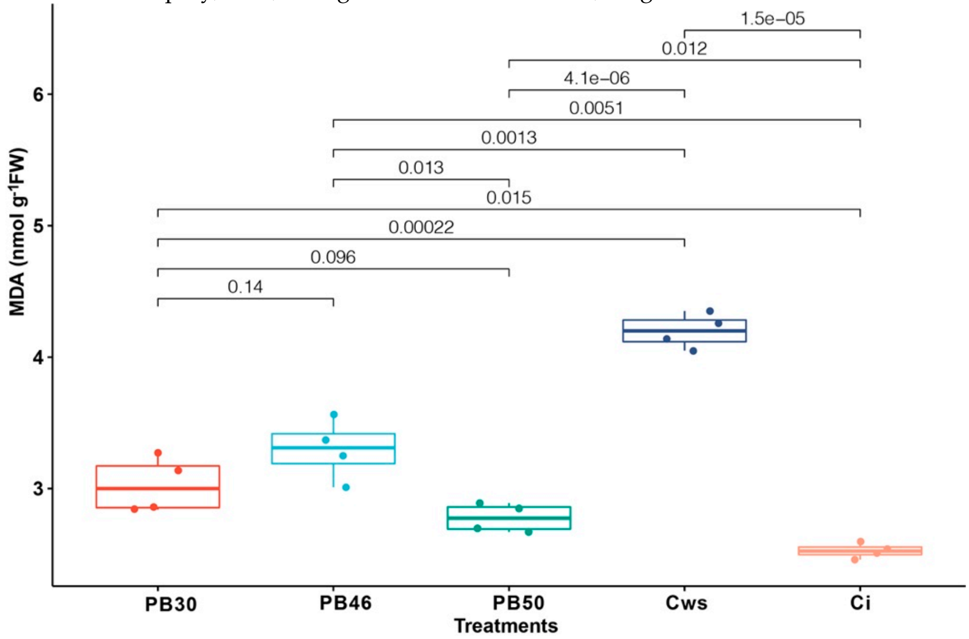

Figure S8: Malondialdehyde (MDA), in rice leaves of different treatment, as well as statistically (ANOVA) significant differences between treatments (ANOVA, p<0.000) after 10 days of drought stress.

Data shown are mean and standard deviation (n = 4). Numbers given above the brackets are P-values indicating statistically significant differences between two treatments according to the Duncan`s test. PB3, drought stress with *B. endophyticus* PB3 foliar spray; PB46, drought stress with *B. altitudinis* PB46 foliar spray; PB50, drought stress with *B. megaterium* PB50 foliar spray; Cws, drought stress control and Ci, irrigated control.

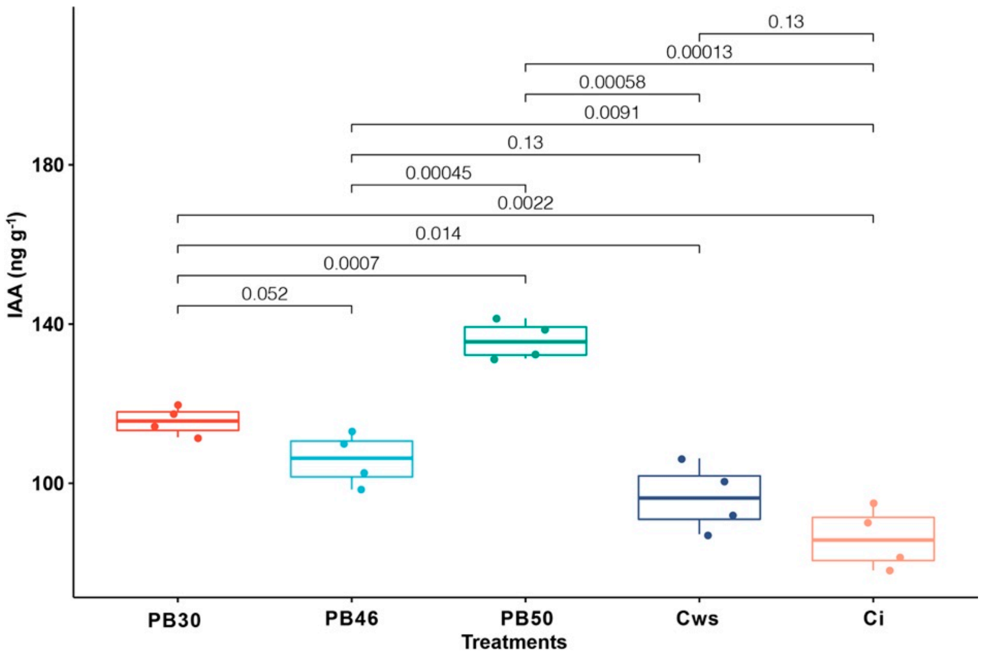

Figure S9: Indole acetic acid (IAA), in rice leaves of different treatment, as well as statistically (ANOVA) significant differences between treatments (ANOVA, p<0.000) after 10 days of drought stress.
Data shown are mean and standard deviation (n = 4). Numbers given above the brackets are P-values indicating statistically significant differences between two treatments according to the Duncan`s test. PB3, drought stress with *B. endophyticus* PB3 foliar spray; PB46, drought stress with *B. altitudinis* PB46 foliar spray; PB50, drought stress with *B. megaterium* PB50 foliar spray; Cws, drought stress control and Ci, irrigated control.

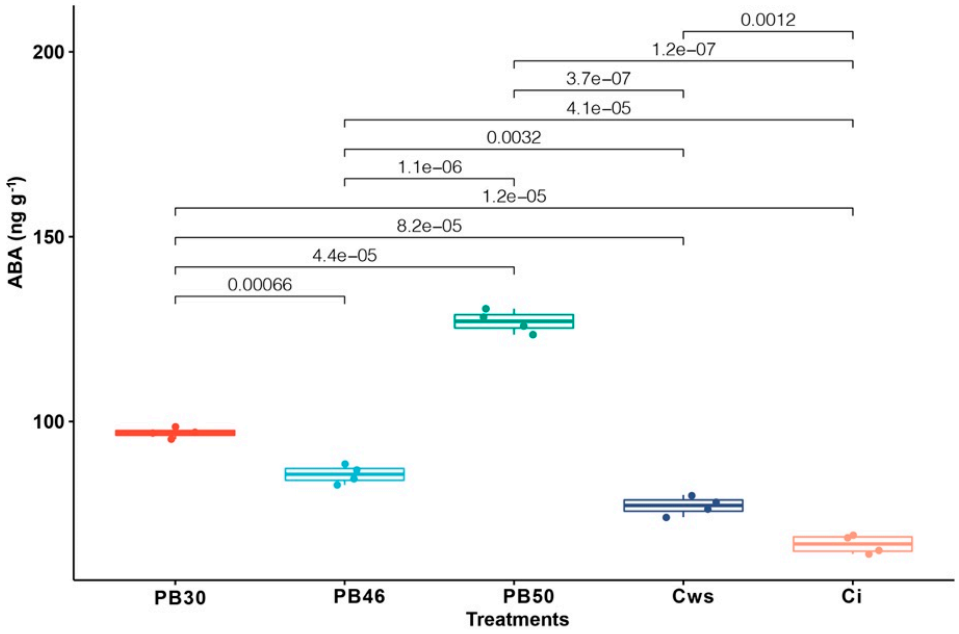

Figure S10: Abscisic acid (ABA), in rice leaves of different treatment, as well as statistically (ANOVA) significant differences between treatments (ANOVA, p<0.000) after 10 days of drought stress.
Data shown are mean and standard deviation (n = 4). Numbers given above the brackets are P-values indicating statistically significant differences between two treatments according to the Duncan`s test. PB3, drought stress with *B. endophyticus* PB3 foliar spray; PB46, drought stress with *B. altitudinis* PB46 foliar spray; PB50, drought stress with *B. megaterium* PB50 foliar spray; Cws, drought stress control and Ci, irrigated control.

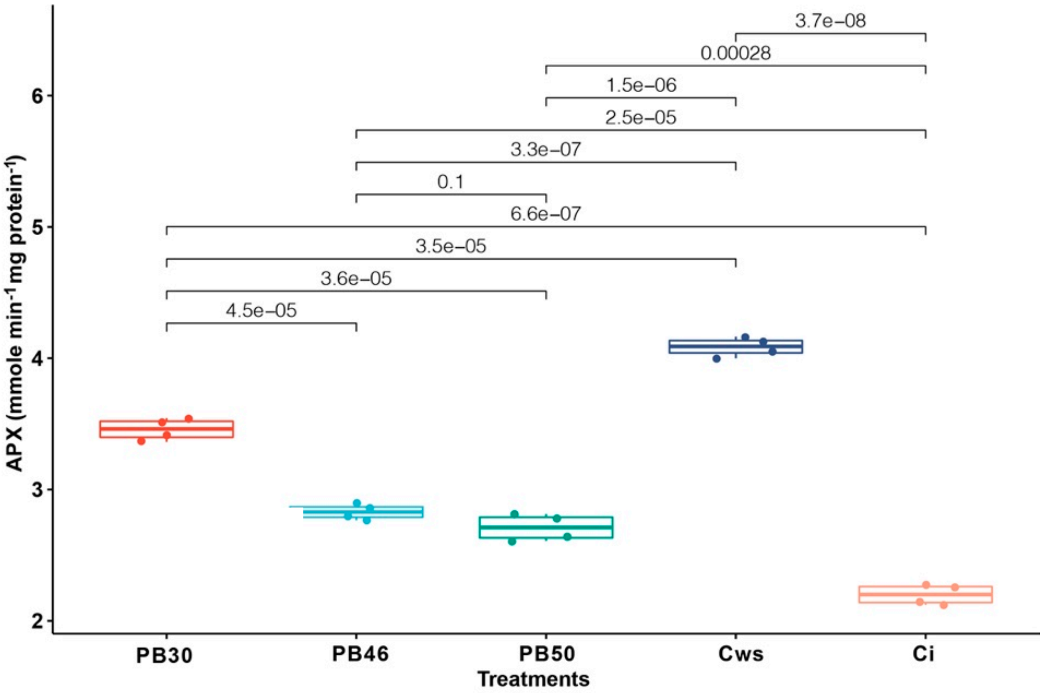

Figure S11: Ascorbate peroxidase (APX) in rice leaves of different treatment, as well as statistically (ANOVA) significant differences between treatments (ANOVA, p<0.000) after 10 days of drought stress.
Data shown are mean and standard deviation (n = 4). Numbers given above the brackets are P-values indicating statistically significant differences between two treatments according to the Duncan`s test. PB3, drought stress with *B. endophyticus* PB3 foliar spray; PB46, drought stress with *B. altitudinis* PB46 foliar spray; PB50, drought stress with *B. megaterium* PB50 foliar spray; Cws, drought stress control and Ci, irrigated control.

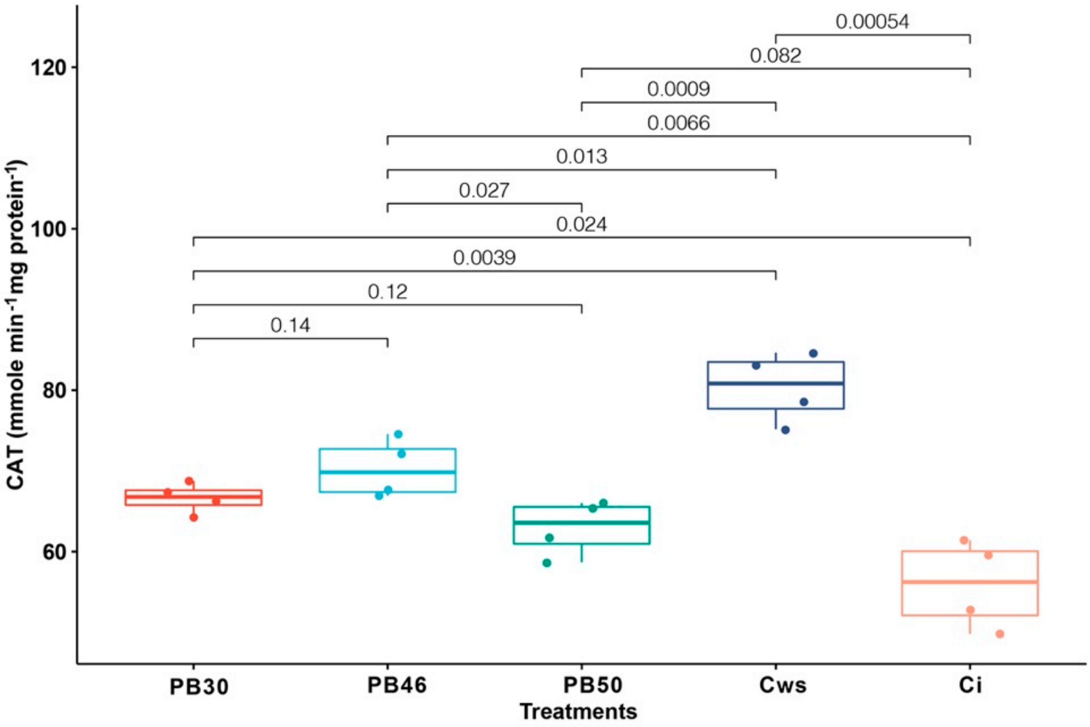

Figure S12: Catalase (CAT) in rice leaves of different treatment, as well as statistically (ANOVA) significant differences between treatments (ANOVA, p<0.000) after 10 days of drought stress.
Data shown are mean and standard deviation (n = 4). Numbers given above the brackets are P-values indicating statistically significant differences between two treatments according to the Duncan`s test. PB3, drought stress with *B. endophyticus* PB3 foliar spray; PB46, drought stress with *B. altitudinis* PB46 foliar spray; PB50, drought stress with *B. megaterium* PB50 foliar spray; Cws, drought stress control and Ci, irrigated control.

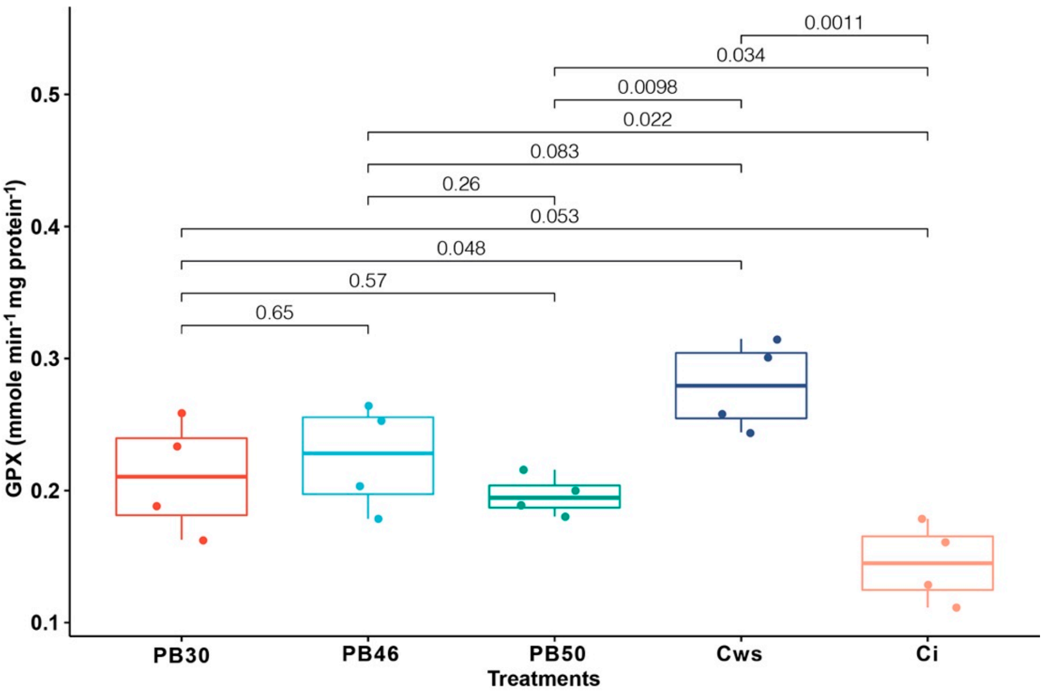

Figure S13: Glutathione peroxidase (APX) in rice leaves of different treatment, as well as statistically (ANOVA) significant differences between treatments (ANOVA, p<0.000) after 10 days of drought stress.

Data shown are mean and standard deviation (n = 4). Numbers given above the brackets are P-values indicating statistically significant differences between two treatments according to the Duncan`s test. PB3, drought stress with *B. endophyticus* PB3 foliar spray; PB46, drought stress with *B. altitudinis* PB46 foliar spray; PB50, drought stress with *B. megaterium* PB50 foliar spray; Cws, drought stress control and Ci, irrigated control.

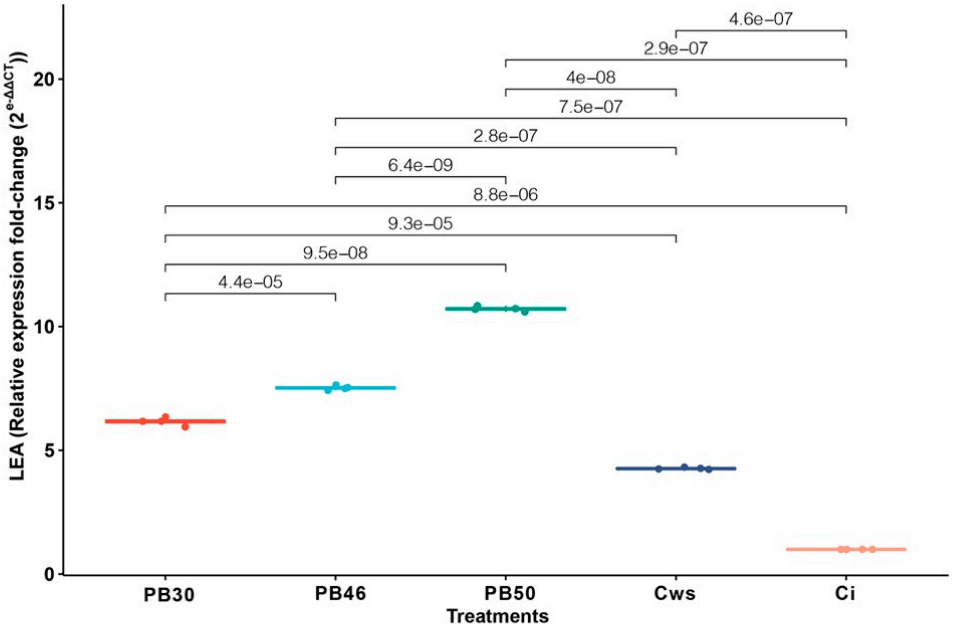

Figure S14: Relative gene expression of *LEA* in rice leaves of different treatment, as well as statistically (ANOVA) significant differences between treatments (ANOVA, p<0.000) after 10 days of drought stress. Data shown are mean and standard deviation (n = 4). Numbers given above the brackets are P-values indicating statistically significant differences between two treatments according to the Duncan`s test. PB3, drought stress with *B. endophyticus* PB3 foliar spray; PB46, drought stress with *B. altitudinis* PB46 foliar spray; PB50, drought stress with *B. megaterium* PB50 foliar spray; Cws, drought stress control and Ci, irrigated control.

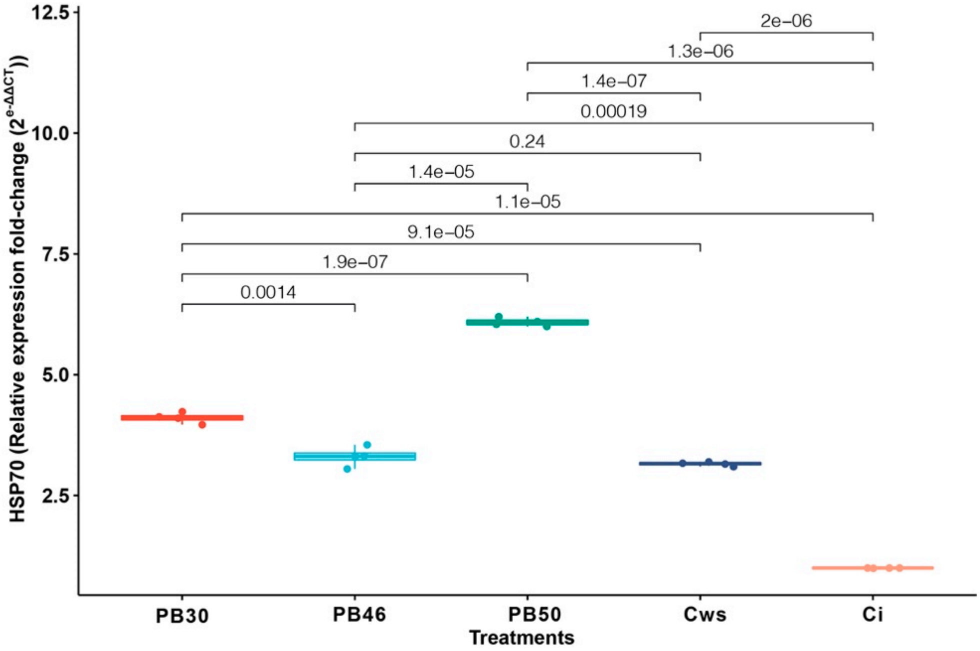

Figure S15: Relative gene expression of *HSP70* in rice leaves of different treatment, as well as statistically (ANOVA) significant differences between treatments (ANOVA, p<0.000) after 10 days of drought stress. Data shown are mean and standard deviation (n = 4). Numbers given above the brackets are P-values indicating statistically significant differences between two treatments according to the Duncan`s test. PB3, drought stress with *B. endophyticus* PB3 foliar spray; PB46, drought stress with *B. altitudinis* PB46 foliar spray; PB50, drought stress with *B. megaterium* PB50 foliar spray; Cws, drought stress control and Ci, irrigated control.

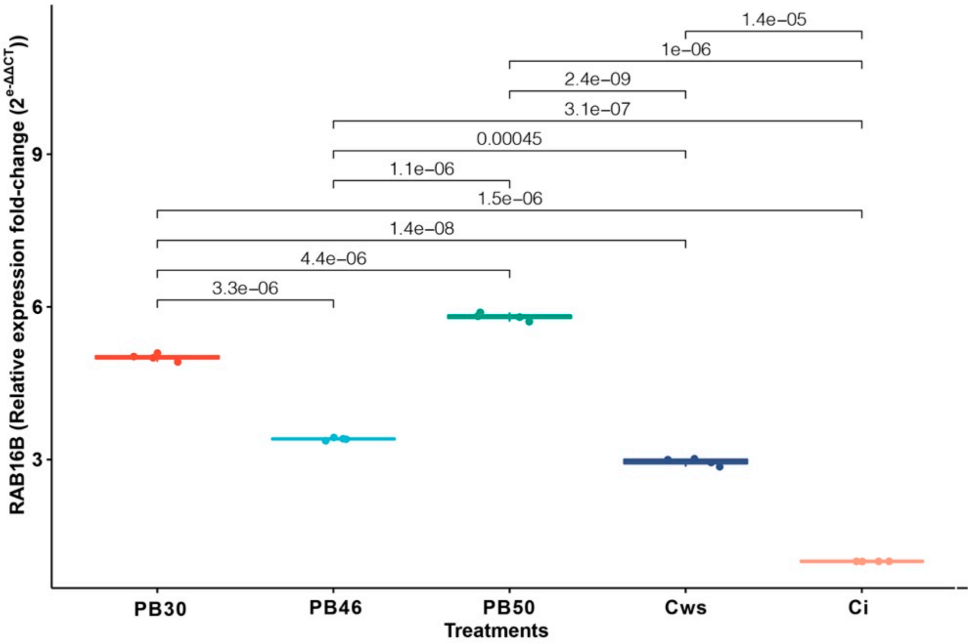

Figure S16: Relative gene expression of *RAB16B* in rice leaves of different treatment, as well as statistically (ANOVA) significant differences between treatments (ANOVA, p<0.000) after 10 days of drought stress. Data shown are mean and standard deviation (n = 4). Numbers given above the brackets are P-values indicating statistically significant differences between two treatments according to the Duncan`s test. T_1, Drought stress with *B. endophyticus* PB3 foliar spray; T_2, Drought stress with *B. altitudinis* PB46 foliar spray; T_3, Drought stress with *B. megaterium* PB50 foliar spray; T_4, Drought stress control and T_5, Irrigated control.

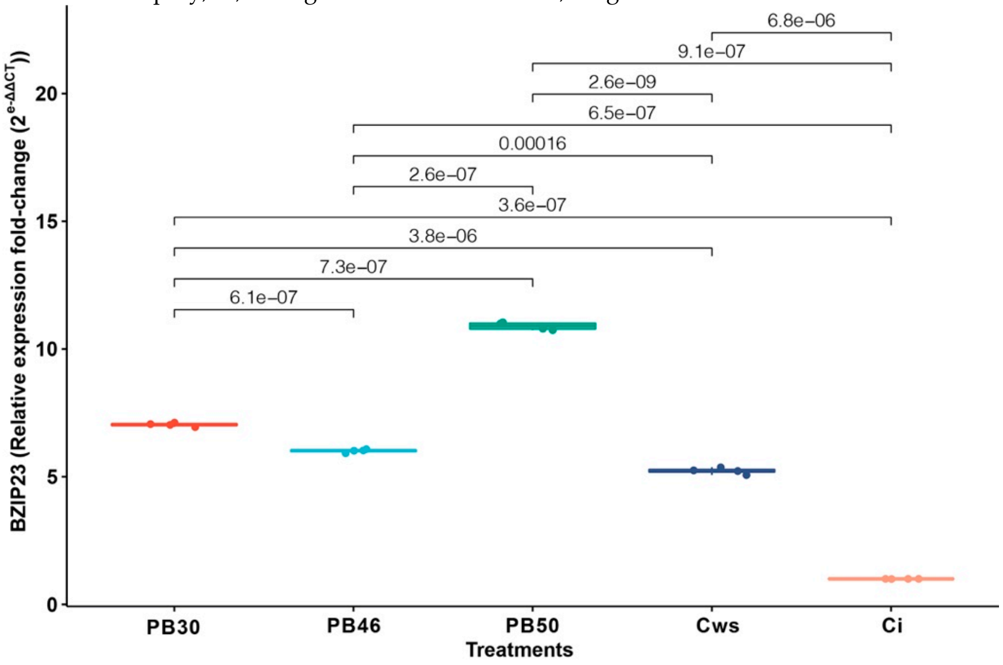

Figure S17: Relative gene expression of *bZIP23* in rice leaves of different treatment, as well as statistically (ANOVA) significant differences between treatments (ANOVA, p<0.000) after 10 days of drought stress. Data shown are mean and standard deviation (n = 4). Numbers given above the brackets are P-values indicating statistically significant differences between two treatments according to the Duncan`s test. PB3, drought stress with *B. endophyticus* PB3 foliar spray; PB46, drought stress with *B. altitudinis* PB46 foliar spray; PB50, drought stress with *B. megaterium* PB50 foliar spray; Cws, drought stress control and Ci, irrigated control.

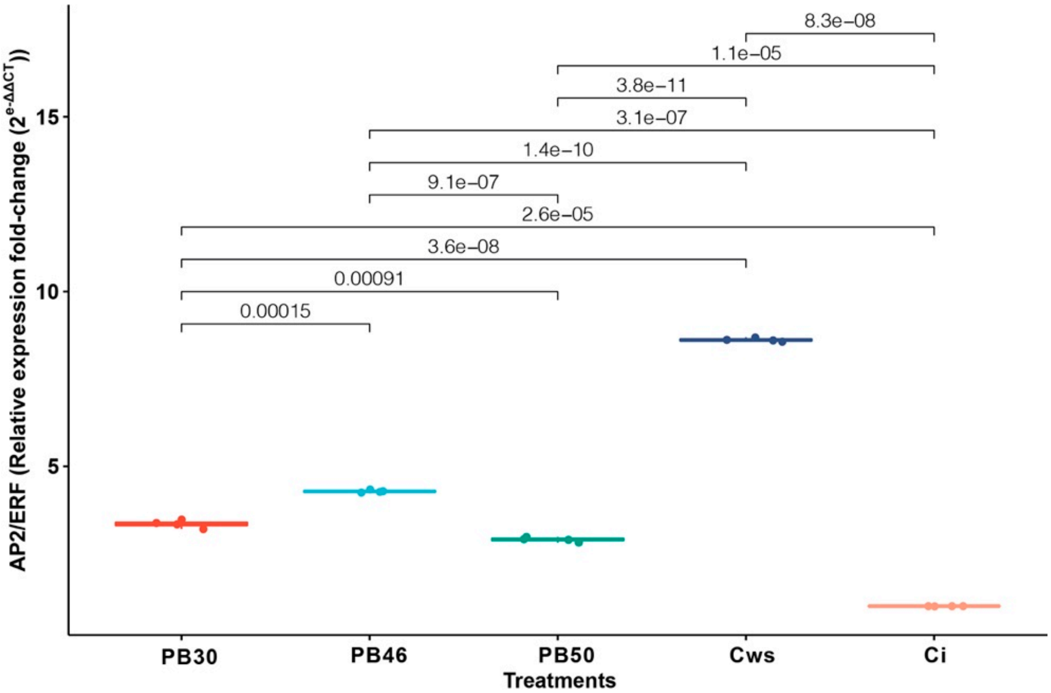

Figure S18: Relative gene expression of *AP2/ERF* in rice leaves of different treatment, as well as statistically (ANOVA) significant differences between treatments (ANOVA, p <0.000) after 10 days of drought stress. Data shown are mean and standard deviation (n = 4). Numbers given above the brackets are P-values indicating statistically significant differences between two treatments according to the Duncan`s test. PB3, drought stress with *B. endophyticus* PB3 foliar spray; PB46, drought stress with *B. altitudinis* PB46 foliar spray; PB50, drought stress with *B. megaterium* PB50 foliar spray; Cws, drought stress control and Ci, irrigated control.

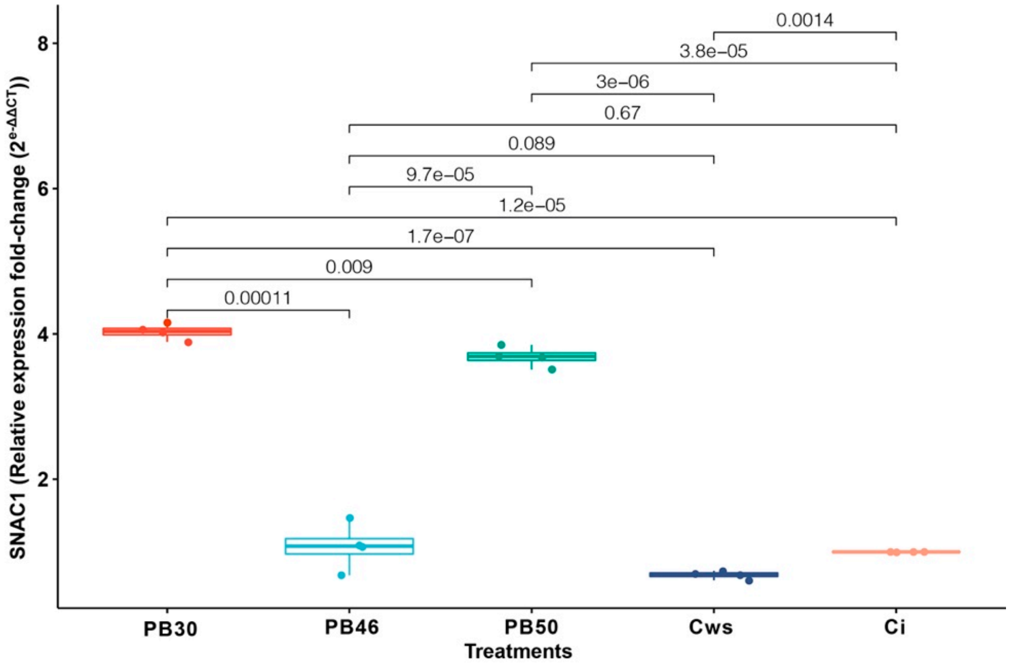

Figure S19: Relative gene expression of *SNAC1* in rice leaves of different treatment, as well as statistically (ANOVA) significant differences between treatments (ANOVA, p <0.000) after 10 days of drought stress. Data shown are mean and standard deviation (n = 4). Numbers given above the brackets are P-values indicating statistically significant differences between two treatments according to the Duncan`s test. PB3, drought stress with *B. endophyticus* PB3 foliar spray; PB46, drought stress with *B. altitudinis* PB46 foliar spray; PB50, drought stress with *B. megaterium* PB50 foliar spray; Cws, drought stress control and Ci, irrigated control.
